# Immune parameters of patients treated with laquinimod, a novel oral therapy for the treatment of multiple sclerosis: results from a double-blind placebo-controlled study

**DOI:** 10.1002/iid3.42

**Published:** 2015-03-04

**Authors:** Mariusz Stasiolek, Ralf A Linker, Liat Hayardeny, Oren Bar Ilan, Ralf Gold

**Affiliations:** 1 Department of Neurology, Polish Mother's Memorial Hospital—Research InstituteLodz, Poland; 2 Department of Neurology, Ruhr-University BochumSt. Josef-Hospital, Bochum, Germany; 3 Teva Innovative Research and Development Group, Teva Pharmaceutical Industries Ltd5 Bazel Street, Petah, Tiqva, 49131, Israel; 4 Department of Neurology, Friedrich-Alexander-University ErlangenGermany

**Keywords:** immunomodulation, laquinimod, monocytes, multiple sclerosis, neuroimmunology

## Abstract

Laquinimod is a novel orally administered drug for the treatment of relapsing remitting multiple sclerosis (RRMS). In this immunological substudy of the phase III Assessment of Oral Laquinimod in Preventing Progression of MS (ALLEGRO) trial, we performed an ex vivo and in vitro analysis of effects exerted by laquinimod on peripheral blood immune cell populations from RRMS patients with a special focus on monocyte phenotype and function. Approximately 100 patients were enrolled following a standardized protocol. Half of the patients received laquinimod and the other half received placebo. Peripheral blood samples were collected prior to commencement of therapy and after 1, 3, 6, 12, and 24 months of continuous therapy. Main lymphocytic and antigen presenting cell fractions were analyzed in peripheral blood mononuclear cells (PBMCs) ex vivo by flow cytometry. The proliferative response of PBMCs to mitogen or recall antigen was assessed in culture experiments. Untouched monocytes were sorted magnetically and cultured under pro-inflammatory conditions. PBMC analysis showed no significant differences of investigated lymphocytic and antigen presenting cell populations over time within each group, or between the two groups. However, the detailed in vitro analysis of monocytes demonstrated a lower level of CD86 expression on monocytes stimulated with LPS in laquinimod patients beginning from the 1st month of treatment. Upon pro-inflammatory stimulation, monocytes obtained from laquinimod treated patients tended to secrete lower levels of the proinflammatory chemokines CCL2 or CCL5. Taken together, in this prospective study, we demonstrate immune modulation but no immunosuppressive biological activity of laquinimod in a large group of MS patients.

## Introduction

Laquinimod (quinolin-3-carboxamide) is a novel, orally administered immunomodulatory drug which was developed for the treatment of relapsing–remitting multiple sclerosis (RRMS). It was selected based on an extensive structure-activity relationship program, which showed a high oral bioavailability, increased potency and a superior safety profile versus other candidates 1. In preclinical studies, laquinimod effectively inhibited disease incidence and relapses of experimental autoimmune encephalomyelitis (EAE) 1–3. The mechanism of action may involve complex immune modulatory processes directed on specific elements of the autoimmune reaction. In a murine model of acute EAE, laquinimod did not reduce the number of B- or T-cells 2. Rather it suppressed Th1 and Th17 cells and induced a Th2/3 shift of the immune response 2–7. There was an associated down-regulation of TNF-α and IL-12 and an up-regulation of TGF-β, IL-4, and IL-10. In parallel, laquinimod reduced the amount of leukocyte infiltration in the central nervous system (CNS) 2,4. Importantly, the prophylactic and therapeutic administration of laquinimod in EAE resulted not only in a lower extent of CNS demyelination, but also in reduced acute axonal damage as well as in functional improvement of callosal axon excitability 5,6. Most remarkably, laquinimod prevented atrophy of ventral horn motor neurons in EAE even when administered after the peak of the disease 6. Additionally, a neuroprotective capacity of laquinimod was suggested by a recent report of our group showing that laquinimod induced a significant and persistent increase in brain derived neurotrophic factor (BDNF) serum levels of MS patients 8. The role of BDNF for its mechanism of action was further confirmed by experiments showing a blunted effect of laquinimod in EAE in mice lacking BDNF expression in myeloid cells and T cells 8. Transfer experiments in the same study also proved a role for monocytes as targets for laquinimod. Another possible mechanism of laquinimod associated neuroprotective activity was demonstrated in the cuprizone model of toxic demyelination. Here, laquinimod treatment prevented demyelination and acute axonal injury independently from the immune response in a mechanism involving direct, CNS intrinsic modulation of NF-kB signaling in astrocytes 9. The local CNS-specific laquinimod mode of action is well in concordance with the results of autoradiography studies showing rapid absorption and distribution of orally administrated ^14^C-laquinimod in the cerebrum, cerebellum, and spinal cord of laboratory animals suffering from EAE but also with an intact blood-brain barrier (BBB) 10.

In inflammatory and autoimmune models other than EAE, laquinimod reduced or prevented clinical manifestations in an experimental model of systemic lupus erythematosus 11. Laquinimod also diminished T-cell responses in experimental models of autoimmune neuritis 3,12. However, laquinimod did not exert any effect on graft survival in a rat heterotopic heart allograft model 13, suggesting a lack of influence on the heterotypic response of an animal to mount a cellular or humoral transplant reaction. Taken together, these data indicate that laquinimod is not an immunosuppressant, but rather acts as an immunomodulator with strong and complex neuroprotective activities.

In MS patients, laquinimod was effective in suppressing MRI-measured disease activity in both phase IIa and IIb clinical trials, and was well tolerated without any opportunistic infection 14. Patients treated with laquinimod exhibited a 44% reduction in the mean cumulative number of active brain lesions as compared with placebo, and a significant decrease in the cumulative number of both Gd-enhancing and new T1 brain lesions 15,16. In turn, two large phase III clinical trials evaluated the effects of a daily dosage of 0.6 mg laquinimod on clinical measures, and MRI measures of permanent axonal damage, as compared to either placebo or interferon-β1a in RRMS patients. The placebo-controlled phase III trial Assessment of Oral Laquinimod in Preventing Progression of MS (ALLEGRO) demonstrated clear effects of laquinimod 0.6 mg in delaying disease progression and on reducing the accumulation of brain-tissue loss, as measured by MRI, together with a favorable safety and tolerability profile 17. The phase III comparison of laquinimod with interferon-beta-1a (BRAVO) trial demonstrated an insignificant effect of laquinimod on disability progression, but significant reductions in brain atrophy versus placebo, again with a very good safety profile 18.

In this immunological substudy of the phase III ALLEGRO trial, we sought to evaluate the immunological effects of laquinimod on PBMC populations and monocyte phenotype/function in RRMS patients. We focused on patients enrolled in Germany and analyzed a panel of cellular and humoral immune functions.

## Methods

### Study design

Prospective longitudinal analysis of changes in PBMC populations and monocyte phenotype and function was performed in patients enrolled in the phase III ALLEGRO trial. Eighty-seven patients gave informed consent to participate in this immunological substudy and were enrolled from multiple sites across Germany (12 ALLEGRO study centers participating in the substudy). Forty-four patients received laquinimod and 43 patients received placebo. There were no significant differences in the main demographic and clinical parameters between the groups of this substudy. For further patients characteristics see Table1. The immunological substudy was approved by the appropriate local ethics committees. Peripheral blood was collected as baseline before the initiation of laquinimod treatment (baseline) and after 1 month (1 m), 3 months (3 m), 6 months (6 m), 12 months (12 m), and 24 months (24 m) of continuous therapy and shipped to the centralized laboratory at the Department of Neurology, Ruhr-University Bochum, St. Josef-Hospital, Bochum, Germany. Only samples received at the laboratory within 24 h of blood collection were processed and included in the analyses. All laboratory personnel were blinded to treatment assignments throughout the study.

**Table 1:** Patients demographic and clinical characteristics at baseline

	Placebo (n = 43)	Laquinimod (n = 44)
Age (mean ± SD)	40.3 ± 8.2	37.9 ± 8.7
Sex (female/male)	33/10	38/6
Years since first symptom (mean ± SD)	6.1 ± 5.6	5.0 ± 5.1
EDSS (mean ± SD)	2.5 ± 1.0	2.1 ± 1.2
Previous DMT (yes/no)	23/20	18/26

### Isolation of peripheral blood mononuclear cells (PBMCs)

Venous blood (20 mL) was drawn into EDTA-containing tubes at individual study sites. Upon receipt of blood samples, peripheral blood mononuclear cells (PBMC) were isolated from anti-coagulated blood by centrifugation on a discontinuous density gradient (Histopaque 1077, Sigma–Aldrich, Inc., St. Louis, USA). The mononuclear cell fraction was washed three times in phosphate buffered saline (PBS), counted and suspended in PBS for flow cytometric analysis and isolation of untouched monocytes.

### Isolation and culture of untouched monocytes

**Figure 1:**
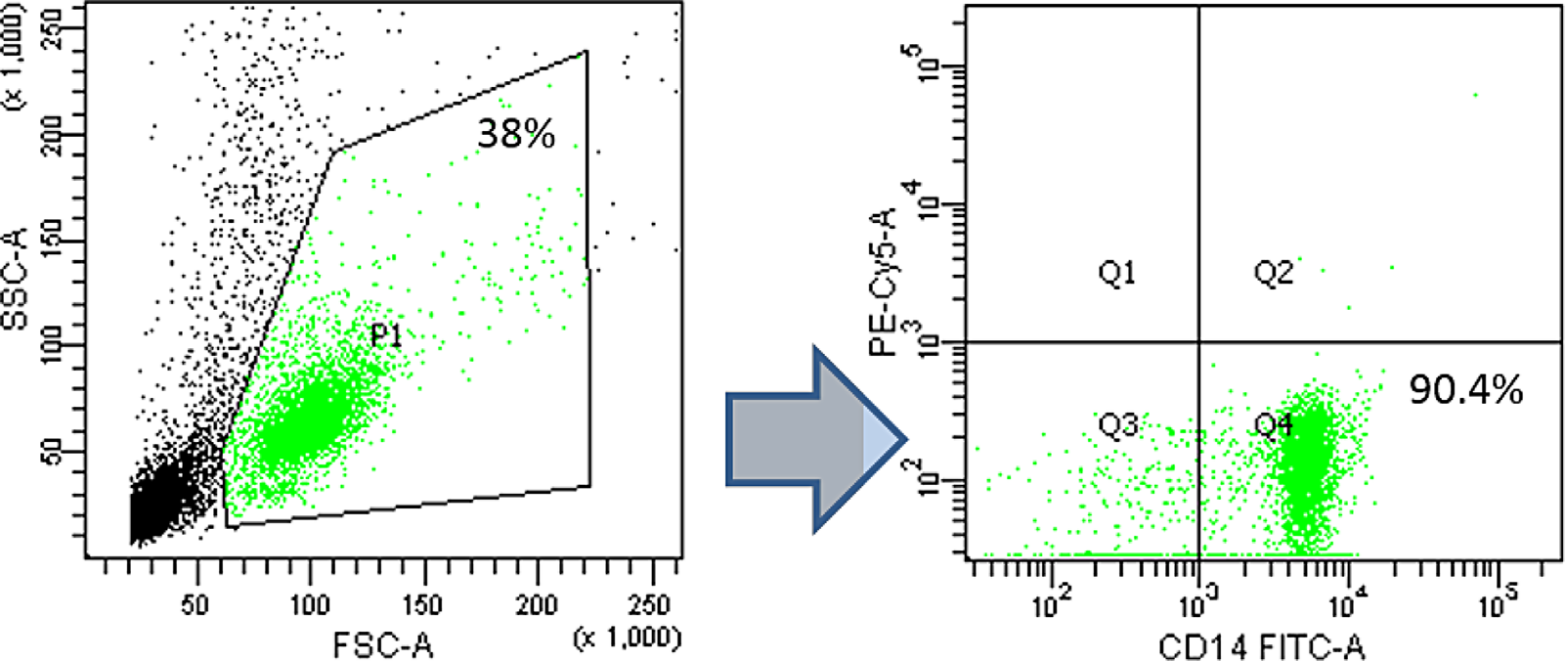
Magnetic sorting of peripheral blood monocytes purity control. Directly after magnetic sorting procedure monocyte fraction was subjected to purity assessment. The percentage of CD14^+^ monocytes was analyzed by Flow Cytometry in a gate encompassing all living cells (P1).

In order to obtain untouched monocytes for in vitro experiments, PBMCs were negatively sorted with the Untouched Monocyte Isolation Kit II (Miltenyi Biotec, Bergisch Gladbach, Germany) in the magnetic field of a MidiMACS® sorter (Miltenyi Biotec), to deplete lymphocytes and granulocytes. The resulting negative fractions typically contained >90% of CD14^+^ monocytes (Fig. 1). Isolated monocytes were plated for 48 h on 96-well culture plates (Nunc, Roskilde, Denmark) at a concentration of 2 × 10^6^/mL in a culture medium containing RPMI 1640, streptomycin 100 µg/mL, penicillin 100 U/mL, 2 mM L-glutamine (Gibco, Life Technologies, Vienna, Austria), and 10% heat-inactivated fetal calf serum (FCS; Boehringer, Mannheim, Germany). For pro-inflammatory stimulation experiments, culture medium was supplemented with 100 U/mL of recombinant interferon-gamma (IFNγ; R&D Systems, USA) or 1 µg/mL lipopolysaccharide (LPS; Sigma–Aldrich, Inc.). After 48 h of culture cells were harvested for flow cytometric analyses and culture supernatants were collected for measurement of inflammatory cytokine and chemokine production.

### Fluorescence-activated cell sorting (FACS) analysis

PBMCs were assessed by multicolor flow cytometry using a FACSCanto II® cytometer and FACSDiva® software (BD Biosciences, San Jose, CA, USA). The major mononuclear cell populations were recognized by staining with fluorochrome conjugated monoclonal antibodies (mAb) specific for key surface antigens: T cells (CD3^+^, CD3^+^CD4^+^, CD3^+^CD8^+^), B cells (CD19^+^), NK cells (CD56^+^CD3^−^), NKT cells (CD56^+^, CD3^+^), monocytes (CD14^+^), mature dendritic cells (CD83^+^). Regulatory T cell populations were identified as CD4^+^CD25^high^FoxP3^+^ lymphocytes. Intracellular staining for Foxp3 was performed on freshly isolated PBMC with the PerCP Anti-Human Foxp3 Staining Set (eBioscience, San Diego, CA, USA) according to manufacturer's protocol. Before fixation, the cells were counterstained with mAbs specific for CD4 and CD25.

After 48 h of culture monocytes were analyzed for HLA-DR and surface expression of the major costimulatory molecules (CD25, CD40, CD69, CD80, CD86). All data were expressed as percent of the whole PBMC fraction.

### PBMC proliferation assays

The relative proliferation of PBMC in the presence of phytohemagglutinin (PHA) or tetanus toxin antigen was assessed. The response of PBMC to mitogen (PHA—5 µg/mL) or recall antigen (tetanus toxin, EMD Millipore, 4 µg/mL) was assessed by plating 10^5^ PBMC with or without stimuli in triplicates. After 48 h of culture at 37°C, 5% CO_2_, wells were pulsed with 0.2 µCi ^3^H-thymidine for 18 h; then cells were harvested and ^3^H incorporation was determined by scintillation counting. Data were calculated as stimulation index, the x-fold increase in cpm incorporation with stimulus as compared to background cpm. Absolute antigen or mitogen-specific responses were reported for each sample as well as the change from baseline.

### Monocyte cytokine secretion assays

Monocyte culture supernatants were analyzed for pro-inflammatory and regulatory cytokine secretion profiles (IL1-β, IL6, IL10, IL12, TNF-α, CCL2/MCP-1, CCL5/RANTES, CXCL8/IL8, CXCL9/MIG, CXCL10/IP10) with the BD Cytometric Bead Array System™ using a FACSCanto II® cytometer and appropriate FlexSets (BD Biosciences).

### Statistical analyses

This study was not designed to test for significant changes in PBMC measures or monocyte responses. The highly heterogeneous nature of the patient population in this study enabled investigators to simply monitor for minor trends that may occur following commencement of laquinimod therapy. Data from the two groups were calculated and visually assessed to demonstrate the outcome results after treatment with laquinimod 0.6 mg versus placebo.

## Results

### Cell surface phenotypes of PBMCs

**Figure 2:**
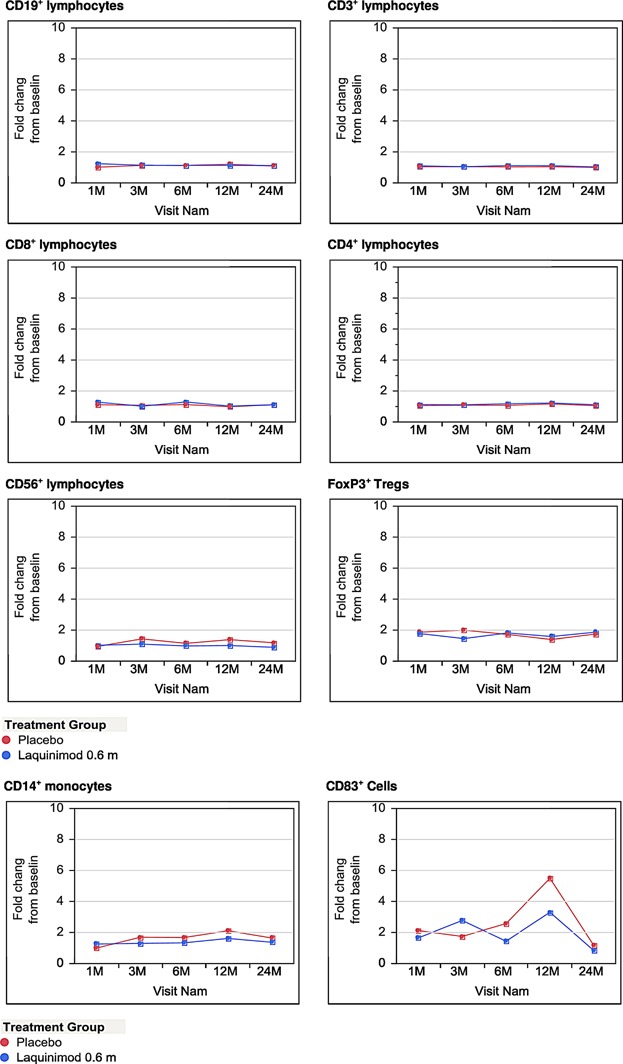
Distribution of main PBMC populations ex vivo. The graphs present percentages of main lymphocyte and antigen presenting cell populations in PBMC fraction freshly isolated from peripheral blood samples obtained from study participants. Due to the very high intra-group variability of particular immune parameters—naturally expected in human study performed on a large group of patients, the results of flow cytometry analysis were expressed as fold change from baseline with base line value for each parameter set as 1.0. Points on charts show the mean values of each subgroup at each time-point.

Preclinical studies in animal models of autoimmunity suggested an immunoregulatory rather than immunosuppressive activity of laquinimod. In order to investigate immunosuppressive versus immunomodulatory effects of laquinimod in MS patients, we conducted a standardized prospective and longitudinal immune-analysis in a subset of individuals participating in the laquinimod phase III trial ALLEGRO. The 2-year long repetitive flow cytometry analysis of the peripheral blood samples obtained from study participants encompassed the major mononuclear cell populations including: CD3^+^CD4^+^ T cells, CD3^+^CD8^+^ T cells, CD4^+^CD25^high^FoxP3^+^ regulatory T cells, CD19^+^ B cells, CD56^+^ NK cells, CD14+ monocytes, and CD83^+^ mature dendritic cells. The measurements were performed in each participant and at each time point on freshly isolated PBMCs with the same protocol. This approach allowed for following individual immune parameters from the beginning to the end of the observation period. After 1, 3, 6, 12, and 24 months of laquinimod treatment, there were no significant quantitative changes in any of the analyzed immune cell populations including CD4^+^ T cells, CD8^+^ T cells, regulatory T cells, B cells, NK cells, NKT cells, monocytes, and mature dendritic cells. There were also no differences in any of the assessed PBMC populations between laquinimod treated individuals and controls receiving placebo at any time point (Fig. 2).

### Proliferative responses of PBMCs

**Figure 3:**
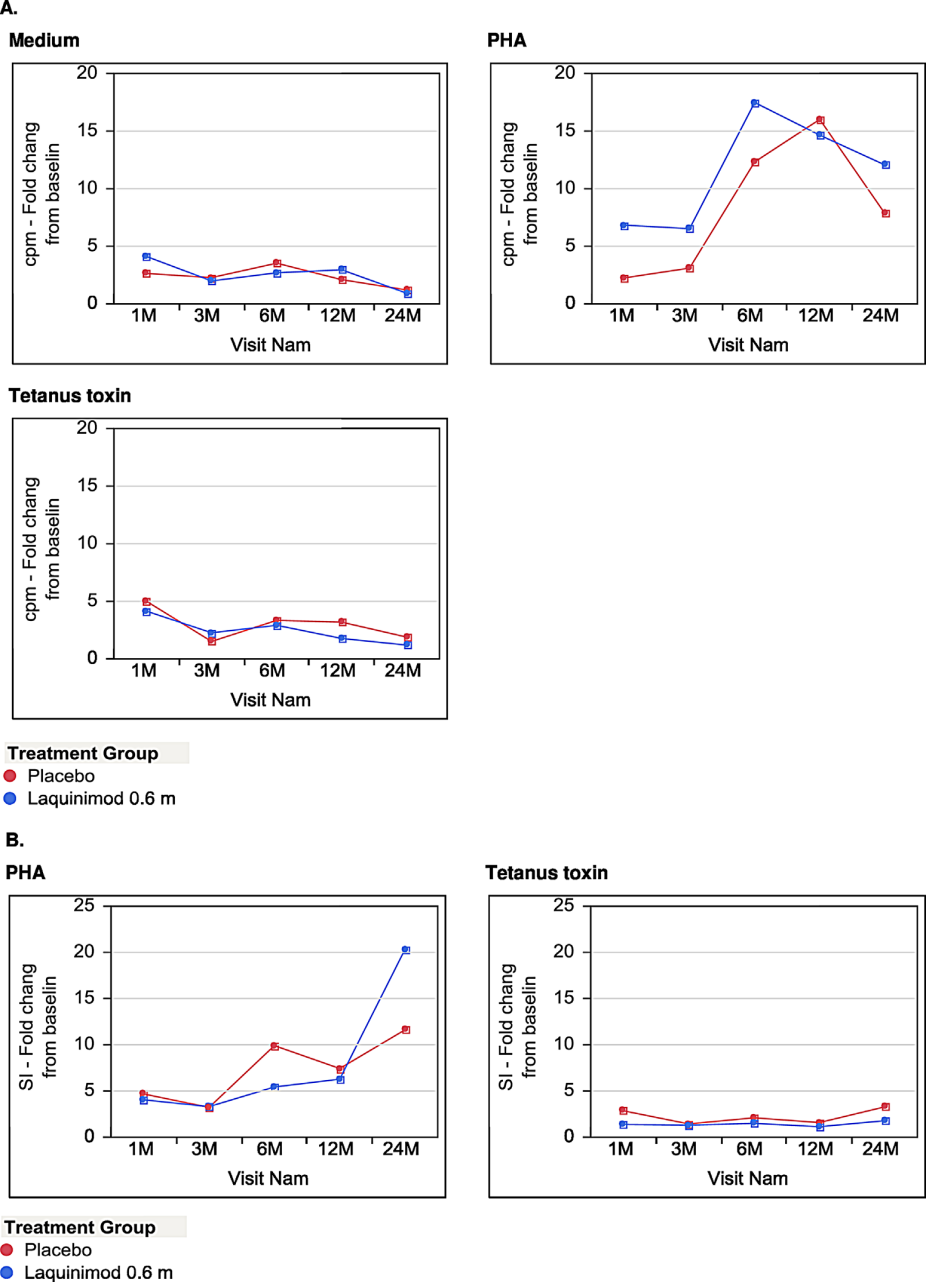
Proliferative responses of PBMC. The graphs present the proliferative response of PBMCs to mitogen (PHA) or recall antigen (tetanus toxin) in vitro. The *y*-axis represents cpm (A) or stimulation index (SI) (B) fold change to baseline values. Points on charts show the mean values of each subgroup at each time-point.

The lack of significant changes in the main peripheral blood immune cell populations does not exclude functional alterations of the immune system under laquinimod administration. As a measure of basic immune function, we thus analyzed the proliferative response of freshly isolated PBMC to mitogen (PHA) or recall antigen (tetanus toxin) in a ^3^H incorporation assay at each study time point (Fig. 3). After 1, 3, 6, 12, and 24 months of laquinimod treatment, there were neither significant differences in antigen-specific nor in mitogen-induced proliferative responses as compared to baseline. Likewise, there were no significant differences in antigen-specific or mitogen-induced proliferative responses between the laquinimod and placebo treated study group over the 2 years of observation. These data further support a non-immunosuppressive mechanism of action of laquinimod in MS patients.

### Response of monocytes to pro-inflammatory stimulation

**Figure 4:**
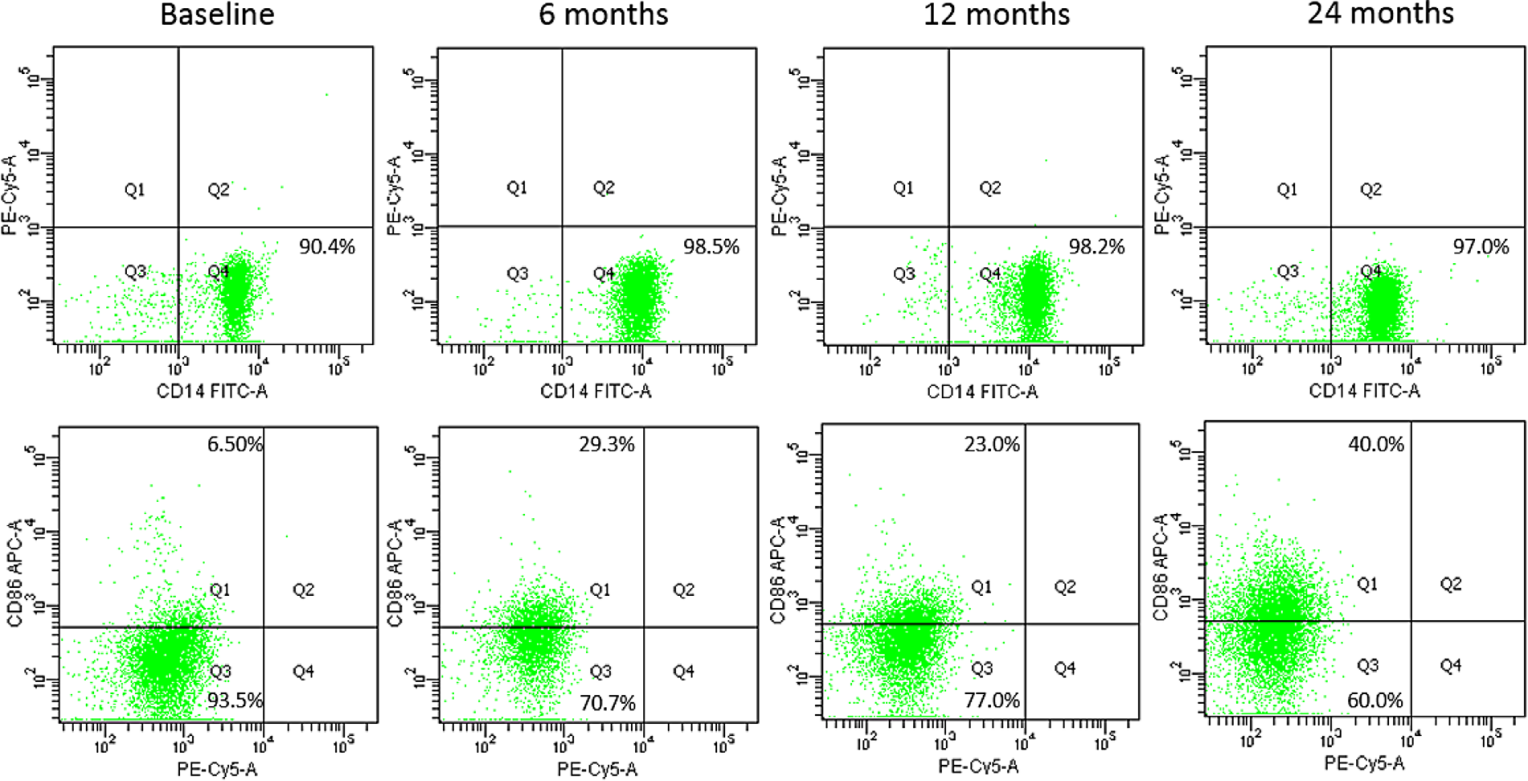
Flow cytometry analysis of monocytes in culture. The plots demonstrate the purity control of monocyte fraction directly after magnetic sorting (upper panel) and CD86 surface expression analysis after 48 h of culture with LPS (lower panel) in the same representative patient over 24 months of the study. The level of specific immunofluorescence was set on the basis of appropriate isotype control staining.

**Figure 5:**
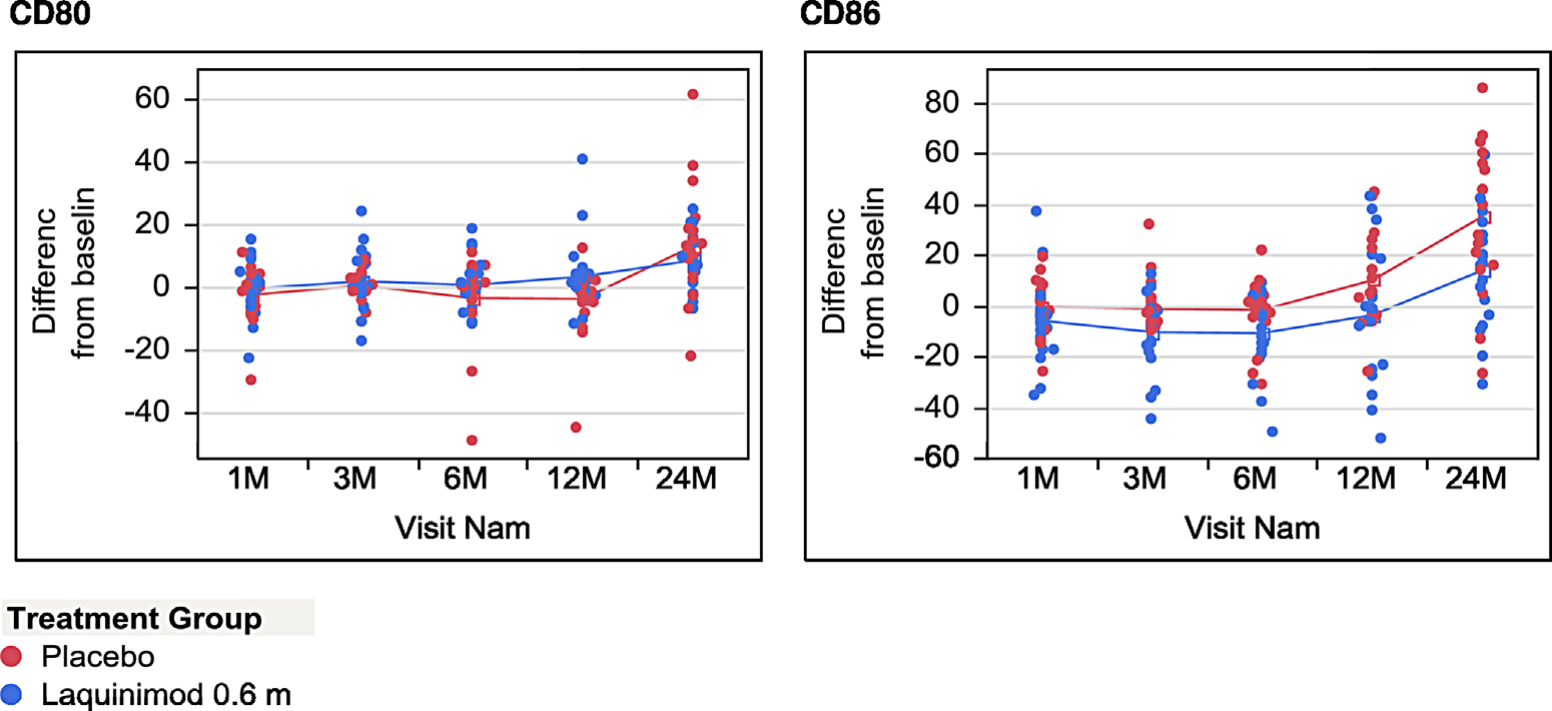
Expression of CD80 or CD86 on monocytes in culture after stimulation with LPS. The graphs present the percentage on CD80 or CD86 positive monocytes stimulated for 48 h with LPS in culture. Due to the high intra-group variability of monocyte phenotype, in order to assess the time related changes in monocyte surface expression of co-stimulatory molecule in laquinimod and placebo treated patients, the results were expressed as ± percentage change from baseline value for each individual patient.

**Figure 6:**
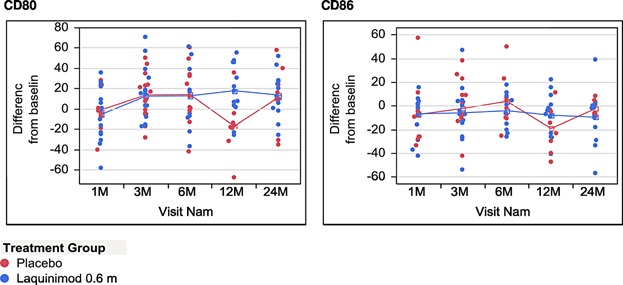
Expression of CD80 or CD86 on monocytes in culture after stimulation with IFNγ. The graphs present the percentage on CD80 or CD86 positive monocytes stimulated for 48 h with IFNγ in culture. Due to the high intra-group variability of monocyte phenotype, in order to assess the time related changes in monocyte surface expression of co-stimulatory molecule in laquinimod and placebo treated patients, the results were expressed as ± percentage change from baseline value for each individual patient.

On the basis of the results of our previous animal experiments indicating monocytes as a possible target of immune regulatory effects of laquinimod, we finally decided to perform a detailed analysis of monocyte phenotypes and cytokine secretion profile in vitro. To this end, monocytes freshly isolated from the peripheral blood of study participants were cultured under pro-inflammatory conditions with IFN-γ or LPS. After 72 h of culture cells were harvested for surface molecule and cytokine expression analysis. Over the course of the study, no influence of laquinimod was observed on the expression of MHC class II molecules neither under IFN-γ nor under LPS stimulation (data not shown). However, beginning form the 1st month of treatment, we observed a lower level of CD86 expression on monocytes stimulated with LPS in laquinimod treated patients (Figs. 4 and 5). This difference was not present in monocytes cultured with IFN-γ (Fig. 6). Importantly, the expression of CD80 as the other main B7 costimulatory molecule was not affected by laquinimod treatment suggesting the presence of CD86-specific as well as stimulation-specific mechanisms of laquinimod action on monocytes.

**Figure 7:**
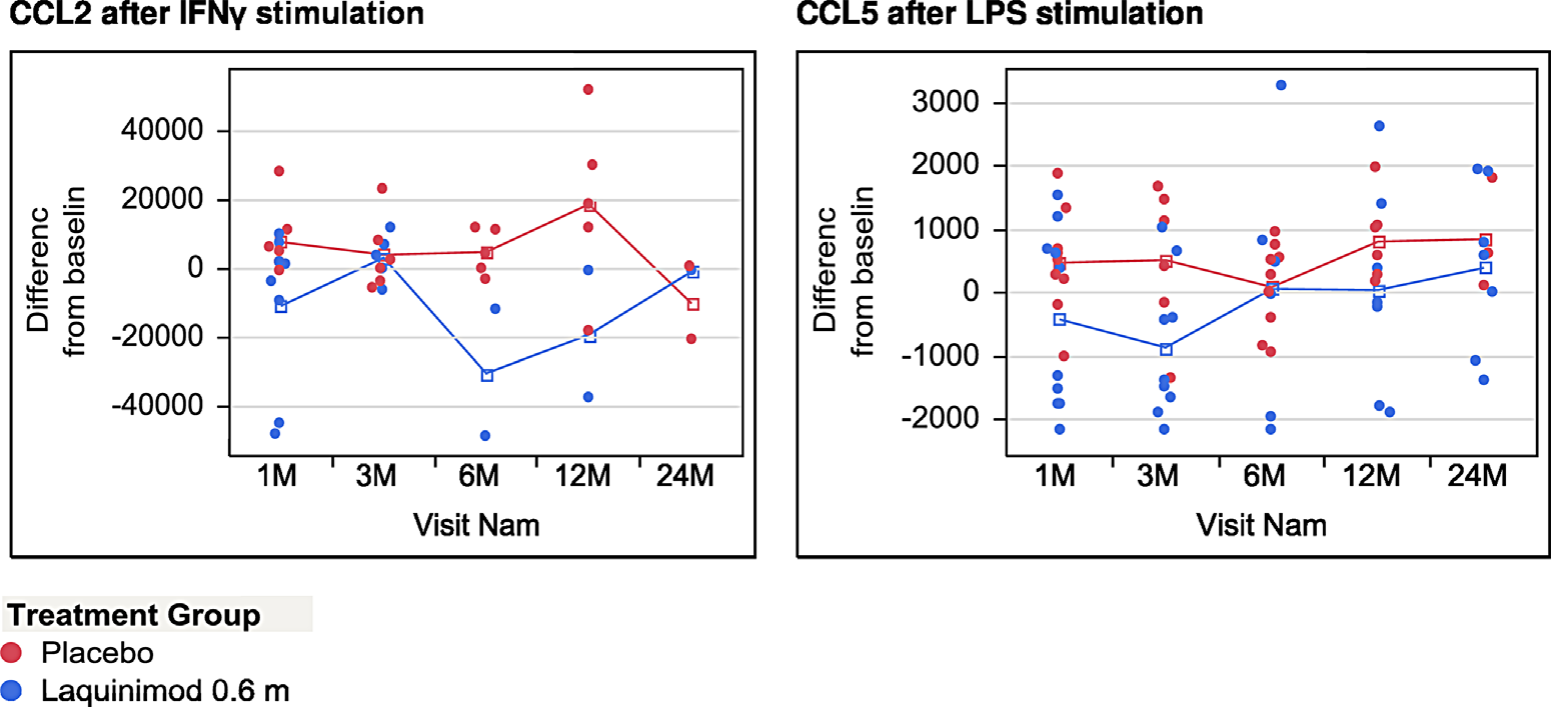
Secretion of CCL2 and CCL5 by monocytes in culture under pro-inflammatory conditions. The graphs present concentration of CCL2 or CCL5 (pg/mL) in monocyte culture supernatants obtained after 48 h of pro-inflammatory stimulation. Due to the exceptionally high intra-group variability of chemokine secretion by monocytes in culture, the results were expressed as ± change from baseline concentration (pg/mL) value for each individual patient.

In addition to the observed in vitro changes on costimulatory molecules, laquinimod treatment also influenced the secretory profile of monocytes cultured in vitro. Throughout the study, the basal secretory profile of unstimulated monocytes in culture did not differ between placebo and laquinimod treated patients (data not shown). However, upon pro-inflammatory stimulation, monocytes obtained from laquinimod treated patients tended to secrete lower levels of proinflammatory chemokines: CCL2 (monocyte chemoattractant protein; MCP-1) and CCL5 (regulated on activation, normal T cell expressed and secreted; RANTES). Interestingly, the down-regulation of CCL2 secretion was observed specifically after IFN-γ stimulation, whereas LPS stimulated monocytes demonstrated a lower production of CCL5 over the course of laquinimod treatment (Fig. 7). We did not observe any effects of laquinimod treatment on the secretion of any other investigated cytokine including IL1-β, IL6, IL10, IL12, TNF-α, CXCL8, CXCL9, and CXCL10 over the course of the study (data not shown).

## Discussion

In our study we prospectively analyzed a broad panel of cellular and humoral immune parameters ex vivo and in vitro in RRMS patients treated continuously for 2 years with laquinimod. The main finding of the analyses performed is a modulatory effect of laquinimod on the immune properties of monocytes stimulated in vitro with various pro-inflammatory factors including LPS and IFNγ, reflecting the immune potential of these cells in vivo.

Already the earliest laquinimod studies in MS showed both protective and disease suppressive effects of this compound in different forms of EAE which is widely regarded as the best animal model of autoimmune CNS demyelination 2,19. The therapeutic effects of laquinimod in EAE were attributed to the anti-inflammatory shift of cytokine secretion (including inhibition of IL-17 production) 2,4,5 and the regulation of CNS migratory properties of both lymphocytic and monocyte/macrophage cells 2,4,5.

Recently, it was shown by our group that adoptive transfer of laquinimod treated monocytes in mice suffering from EAE ameliorated the clinical course of the disease in a mechanism most probably dependent on a monocyte shift towards a regulatory and neuroprotective immune profile associated with BDNF secretion 8. Taking in consideration the immune cell independent, astrocyte specific beneficial effects of laquinimod in the cuprizone demyelination model, our findings suggest monocytes alternatively or complementary to astrocytes as cellular carriers of the laquinimod-associated neuroprotective capacity 8,9. Moreover, the results of a human leukocyte gene expression study indicated monocytes as one of the main targets of the immunoregulatory action of laquinimod 20. Accordingly, in our in vitro analysis we decided to prospectively investigate the influence of laquinimod treatment on monocyte responses to different proinflammatory stimuli. The in vitro assessment of costimulatory profiles repeatedly showed lower levels of LPS stimulated CD86 expression in monocytes isolated from the peripheral blood of laquinimod treated patients, beginning already from the 1st month of the treatment. Interestingly, this difference was not present in monocytes cultured with IFN-γ. Additionally, under pro-inflammatory conditions applied in our experiments (LPS or IFN-γ), we did not observe any differences in the expression of CD80 between monocytes obtained from laquinimod and placebo treated patients. In our opinion, these results suggest the existence of a subtle immuno-regulatory action of laquinimod directed on monocytes which is specific for particular costimulatory mechanisms and dependent on the mode of pro-inflammatory stimulation. Of great importance seems to be the differential influence of laquinimod on the expression of particular members of the B7 family of costimulatory molecules. Due to their functional properties and their kinetics of expression, CD80 and CD86 are considered to play different roles in various phases of the immune reaction: CD86 delivering the main costimulatory signal in the most early stage and CD80 being expressed later after a previous pro-inflammatory stimulation 21,22. Thus, the selective down-regulation of LPS but not IFN-γ stimulated CD86 expression by monocytes may represent a central regulatory mechanism underlying the influence of laquinimod on APCs and in consequence on the putative auto-immune reaction in MS as a whole. This may be further supported by the influence of laquinimod treatment on chemokine secretion by monocytes under inflammatory conditions in vitro. In laquinimod treated patients, the down-regulation of CD86 expression on monocytes upon LPS stimulation was accompanied by a lower production of CCL5, whereas monocytes stimulated in vitro with IFN-γ were characterized by a lower ability to secrete CCL2 with no differences in CCL5 secretion as compared to the placebo group. CCL2 is secreted by monocytes upon proinflammatory stimulation to recruit additional monocytes, memory T cells, and DCs to the site of inflammation. Similarly, CCL5, a member of the IL-8 super-family of cytokines, is a selective attractant for memory T lymphocytes and monocytes 23,24. Both chemokines may play a crucial role in the recruitment and activation of inflammatory monocytes and other pathogenic immune cells to the central nervous system in MS and EAE 25. In active MS lesions, CCL2 was shown to be expressed by monocyte/macrophage cells and astroglia whereas CCL5 production was observed predominantly in non-immune cells, that is, astrocytes and endothelial components 26,27. Importantly, both CCL2 and CCL5 were implicated as important factors regulating monocyte and lymphocyte BBB transmigration in MS 28–30. In agreement with our results showing the influence of laquinimod on chemokine secretion in humans, laquinimod treatment abrogated the CNS entry of pro-inflammatory monocytes in EAE in a mechanism involving the inhibition of local CCL2 production 31. Most interestingly, an analysis of the secretion profile of glial cells in culture showed that laquinimod effects are depended on the glial cell type and the stimulus applied. In primary microglial cultures, laquinimod increased the TNFα-induced CCL5 secretion, whereas such effects were not observed after LPS stimulation. In primary astrocyte cultures stimulated with IL1β alone or in combination with IFNγ, laquinimod strongly reduced the secretory reaction except for CCL5 production, which was increased by laquinimod as in the case of microglial cells 9. The opposite effects on CCL5 secretion after pro-inflammatory stimulation in glial cells and peripheral blood monocytes may suggest that laquinimod acts on immune cell trafficking at multiple levels including the peripheral immune system, the BBB and finally particularly CNS structures.

Recently Jolivel et al. 32 published an elegant experimental study analyzing the influence of laquinimod on DCs. In their experiments, the authors presented a profound effect of laquinimod on the functional properties of DC including cytokine and chemokine secretion and an immuno-regulatory action both in EAE and in MS patients. Importantly, one of the crucial findings of this study was a modulatory influence of laquinimod on CD86 expression on DCs. Both in splenic DCs of EAE mice and in conventional DC from the peripheral blood of MS patients, laquinimod treatment was associated with an increased CD86 expression. Yet, the in vitro experiments with human monocyte derived DCs showed that laquinimod inhibited LPS stimulated CD86 expression, which stays perfectly in line with our prospective 2-year long ex vivo observation. Additionally, similarly to our data Jolivel et al. did not observe any changes in HLA-DR expression in parallel to the CD86 modulatory effects of laquinimod. These results on DC together with our monocyte data imply that the immune-regulatory effects of laquinimod are cell-type specific and may involve a vast spectrum of antigen presenting cells. Such assumptions are confirmed by the results of a study in EAE demonstrating that the decrease of T cell encephalitogenicity in laquinimod treated mice was dependent on changes in APC subsets including the induction of regulatory monocyte and DC subpopulations 7.

In contrast to the in vitro findings, the ex vivo measurements encompassing all the main peripheral blood lymphocytic populations (B cells, T cells, NK cells, NKT cells, Tregs) as well as monocytes and mature CD83^+^ DCs revealed no differences between the laquinimod and placebo treated groups over the 2 years of observation. Additionally, during the study we did not observe any effects of laquinimod treatment on the proliferative response of PBMCs either to mitogen or antigen-specific stimulation in vitro. These findings clearly show that longitudinal treatment with laquinimod does not exert any major, unspecific immunosuppressive influence on the crucial elements of patient's cellular immunity at any time point of the therapy, beginning from the 1st till the 24th month. Hence, we here provide a unique set of immune-safety data for the long-term treatment of MS patients with laquinimod. During the preparation of this manuscript, the results of a complementary study were published which solely focused on ex vivo analyses of immune cells from ALLEGRO study participants in the US 33. In full agreement with the ex vivo part of our study, there was also no effect of laquinimod on the main peripheral blood immune cell populations in the American MS patient population. These results further support the fine immune modulatory but not immunosuppressive biological activity of laquinimod in humans. Importantly, the laquinimod associated regulatory mechanisms may be directed on particular immune cell populations (especially antigen presenting cells) and may, with great probability, be organ- and disease-specific.

## Author Contributions

MS and RL planned and performed all the experimental procedures, LH designed the study and wrote the paper, OB analyzed the experimental data and performed the statistical analysis, RG designed the study and wrote the paper. The study was funded by Teva Pharmaceutical Industries Ltd, Israel.

## Conflict of Interest

LH and OB are employees of Teva Pharmaceutical Industries Ltd, Israel.
